# Supplementary material for: PGRP-LB: An Inside View into the Mechanism of the Amidase Reaction
Source: Int J Mol Sci. 2021 May 7;22(9):4957. doi: 10.3390/ijms22094957 (PMC8124813; doi:10.3390/ijms22094957)
Supplement: Supplementary file 1 [file ijms-22-04957-s001.zip › ijms-1197069-supplementary.pdf]

SUPPLEMENTARY INFORMATION

PGRP-LB: an inside view into the mechanism of the amidase reaction

Julien Orlans [1,2], Carole Vincent-Monegat [1], Isabelle Rahioui [1], Catherine Sivignon [1], Agata Butryn [2,3], Laurent Soulère [4], Anna Zaidman-Remy [1], Allen M. Orville [2,3], Abdelaziz Heddi [1], Pierre Aller [2,3], Pedro Da Silva [1]

[1]Univ Lyon, INSA Lyon, INRAE, BF2I, UMR 203, 69621 Villeurbanne, France
[2]Diamond Light Source, Harwell Science and Innovation Campus, Didcot, Oxfordshire, OX11 0DE, United Kingdom
[3]Research Complex at Harwell, Rutherford Appleton Laboratory, Didcot, Oxfordshire, OX11 0FA, United Kingdom
[4]Univ Lyon, INSA Lyon, Université Claude Bernard Lyon 1, CPE Lyon, UMR 5246, CNRS, ICBMS, Institut de Chimie et de Biochimie Moléculaires et Supramoléculaires, Bât. E. Lederer, 1 rue Victor Grignard F-69622 Villeurbanne, France

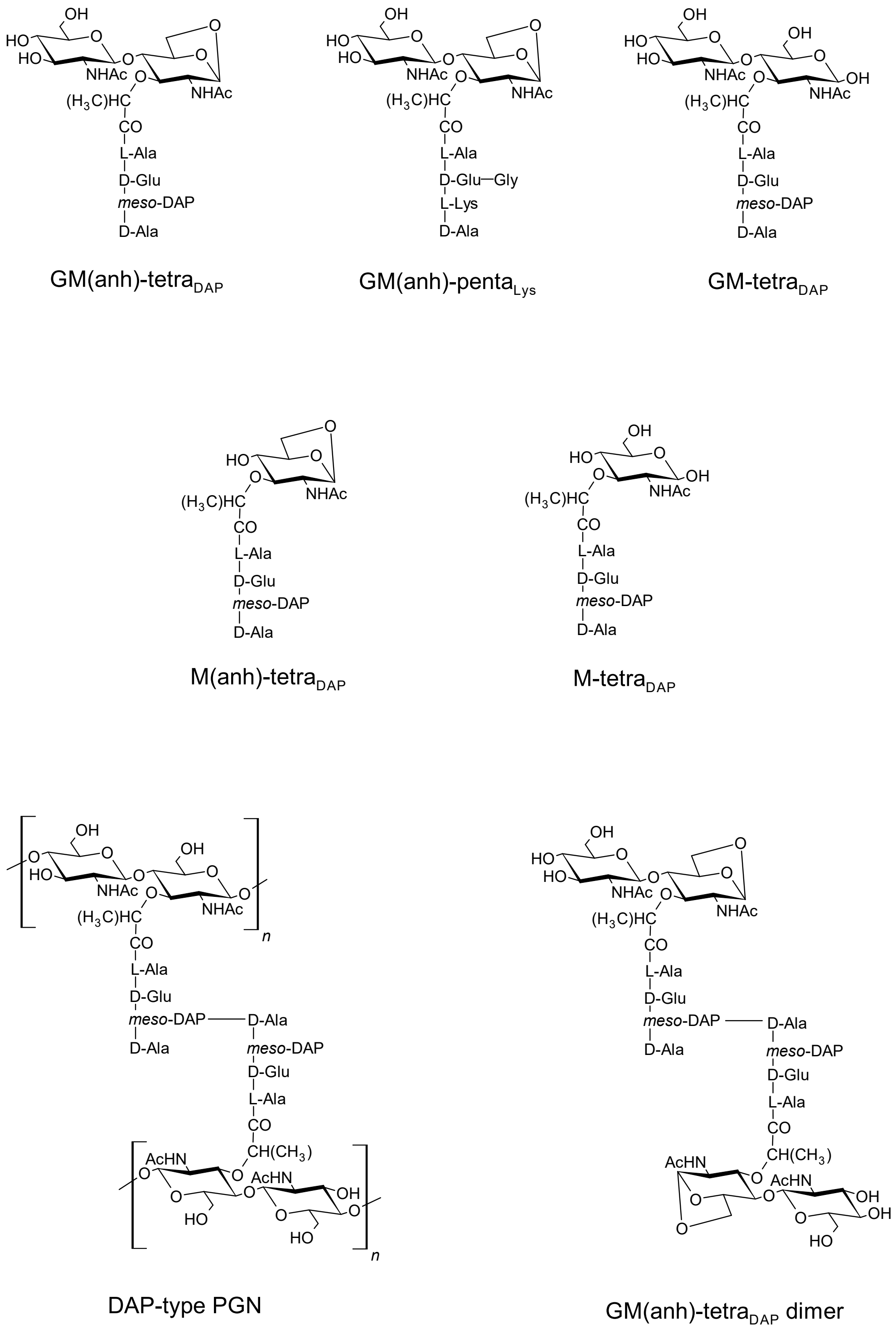

Figure S1. Schematic representation of the *E. coli* PGN and muropeptides used in this study.

Table S1. HPLC retention times of all muropeptides substrates used in this work.

	Enzymatic reaction	Retention time HPLC run 1 (min)*	Retention time HPLC run 2 (min)*
GM(anh)-tetra$_{DAP}$ (TCT)	Substrate: *E. coli* PGN polymeric<br>300 mM sodium acetate pH 4.5<br>0.5 mM MgCl2<br>50 µg SltY	29 (A)	71 (C)
GM(anh)-tetra$_{DAP}$ dimer	Substrate: *E. coli* PGN polymeric<br>300 mM sodium acetate pH 4.5<br>0.5 mM MgCl2<br>50 µg SltY	43 (A)	91 (C)
GM(anh)-penta$_{Lys}$	Substrate: *M. luteus* PGN polymeric<br>300 mM sodium acetate pH 4.5<br>0.5 mM MgCl2<br>50 µg SltY	34 (A)	88 (C)
GM-tetra$_{DAP}$	Substrate: *E. coli* PGN polymeric<br>25 mM potassium phosphate pH 6.5<br>0,25 mM MgCl2<br>20 µL Mutanolysine	20 / 24 (B)	54 / 59 (C)
M(anh)-tetra$_{DAP}$	Substrate: GM(anh)-tetra$_{DAP}$<br>20 mM HEPES buffer pH 7.4<br>50 mM NaCl, 0.5 mM substrate<br>20 µg NagZ	23 (A)	75 (C)
M-tetra$_{DAP}$	Substrate: GM-tetra$_{DAP}$<br>20 mM HEPES buffer pH 7.4<br>50 mM NaCl, 0.5 mM substrate<br>20 µg NagZ	15 / 19 (B)	49 / 56 (C)

*HPLC conditions:
Column: Jupiter® 5 µm C18 300 Å, LC Column 250 x 4.6 mm
A: Elution with 90/10 50 mM sodium phosphate pH 4.5 / 25% MeOH, and application of a linear gradient of 25% MeOH (from 10 to 85%) between 0 and 40 min, at a flow rate of 0.5 ml/min; detection at 210 nm
B: Elution with 100/0 50 mM sodium phosphate pH 4.5 / 25% MeOH, and application of a linear gradient of 25% MeOH (from 0 to 40%) between 0 and 20 min then (from 40 to 85%) between 20 and 25 min, at a flow rate of 0.5 ml/min; detection at 210 nm
C: Elution with 0,4% TFA, and application of a linear gradient of 25% MeOH (from 0 to 100%) between 20 and 105 min, at a flow rate of 0.5 ml/min; detection at 210 nm

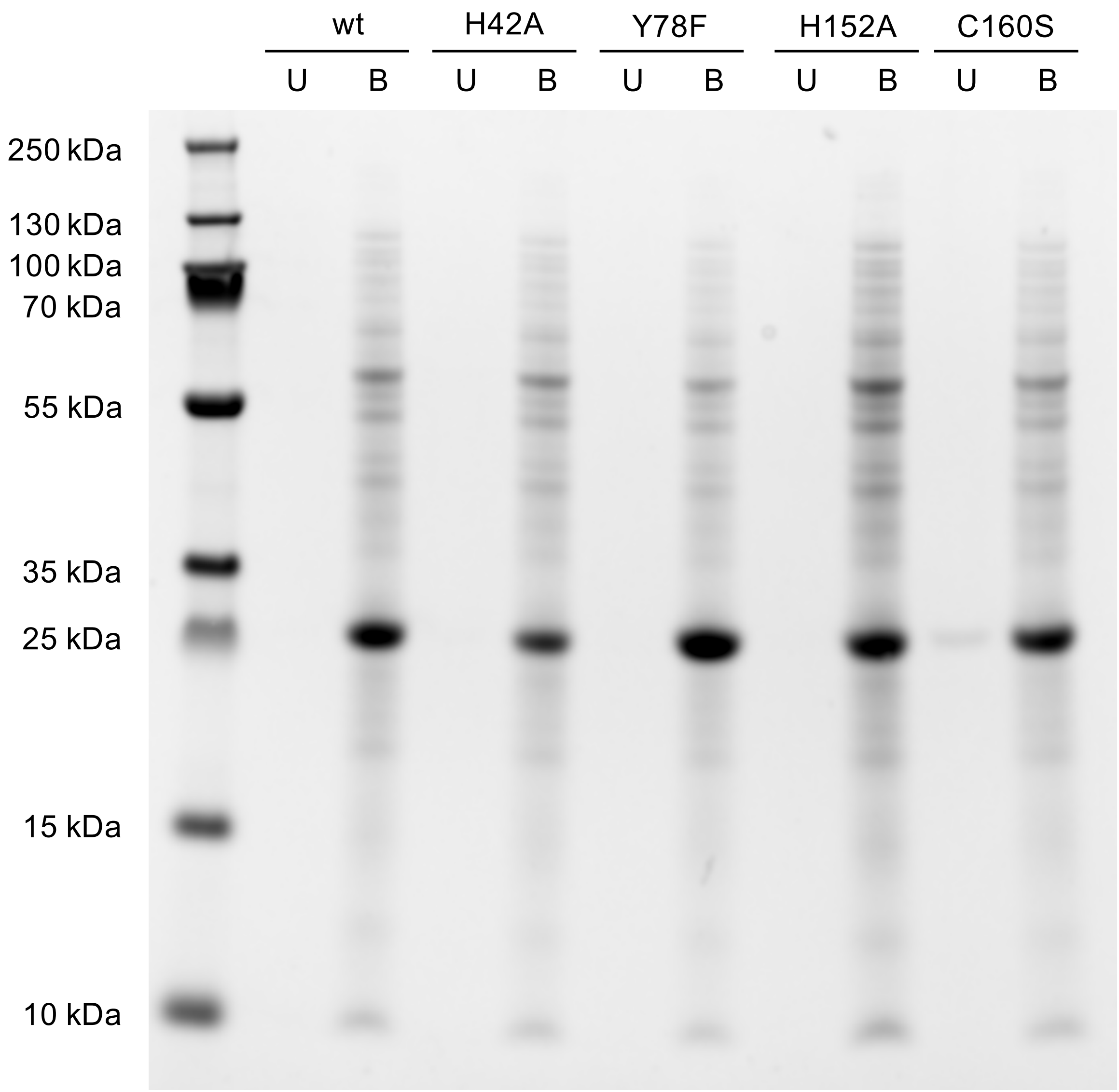

Figure S2. Binding assay of *Drosophila* PGRP-LB$^{PA/PC}$ wild type and its mutants to insoluble *E. coli* polymeric PGN. After incubation with the PGN, the samples are centrifuged, and the supernatant (unbound fraction) is separated from the pellet (bound fraction). Fractions are separated on an SDS-PAGE and proteins are stained by Coomassie. U = unbound; B = bound.

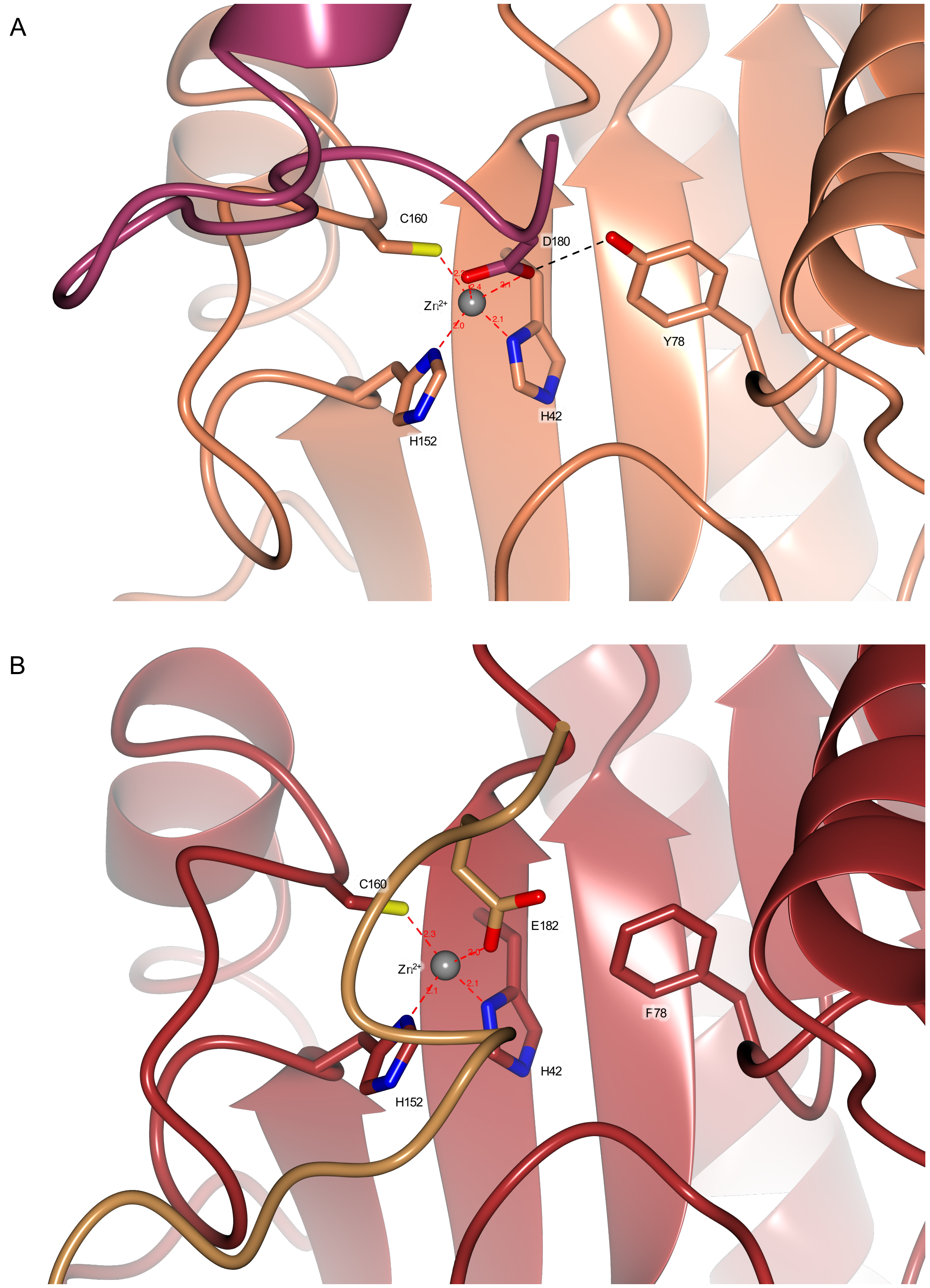

Figure S3. Crystal packing at the Zn^{2+} coordination in *Drosophila* PGRP-LB$^{PA/PC}$ wild-type and mutant PGRP-LB$^{PA/PC}$_Y78F. (**A**) PGRP-LB$^{PA/PC}$ wild-type main chain is colored in coral and the symmetry-related molecule in maroon. (**B**) PGRP-LB$^{PA/PC}$_Y78F main chain is colored in red and the symmetry-related molecule in brown. Hydrogen bonds are represented in black doted lines, red doted lines show the Zn^{2+} (grey ball) chelation. The structures figures were made using CCP4mg.

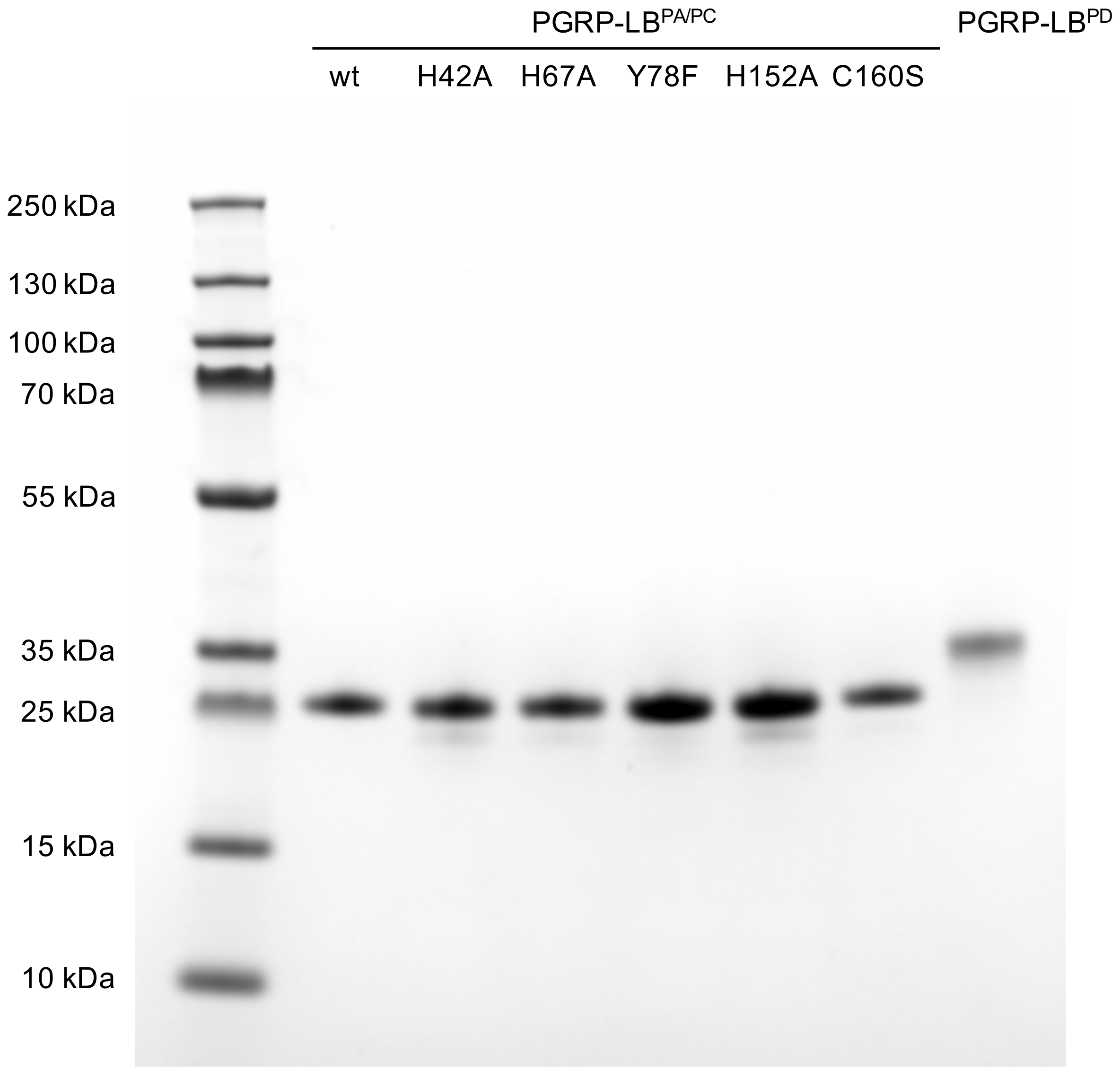

Figure S4. SDS-PAGE of the purified *Drosophila* PGRP-LB isoforms and their mutants used in this study.
